# Seroprevalence of binding and neutralizing antibodies against 18 adeno-associated virus types in patients with neuromuscular disorders

**DOI:** 10.3389/fimmu.2024.1450858

**Published:** 2024-09-27

**Authors:** Xiaoyan Wang, Patrick Julian Klann, Ellen Wiedtke, Yumi Sano, Nico Fischer, Lisa Schiller, Anna Elfert, Anne-Katrin Güttsches, Ute Weyen, Dirk Grimm, Matthias Vorgerd, Wibke Bayer

**Affiliations:** ^1^ Institute for Virology, University Hospital Essen, University Duisburg-Essen, Essen, Germany; ^2^ Berufsgenossenschaftliche-Kliniken Bergmannsheil, University Hospital, Heimer Institute for Muscle Research, Ruhr-University Bochum, Bochum, Germany; ^3^ Department of Infectious Diseases/Virology and Microbiology, Section Viral Vector Technologies, BioQuant, Heidelberg University, Heidelberg, Germany; ^4^ German Center for Infection Research (DZIF) and German Center for Cardiovascular Research (DZHK), Partner Site Heidelberg, Heidelberg, Germany

**Keywords:** adeno-associated virus, viral vector, gene therapy, neuromuscular disease, seroprevalence, neutralizing antibodies, binding antibodies

## Abstract

High levels of pre-existing antibodies are a major challenge for the application of viral vectors since they can severely limit their efficacy. To identify promising candidates among adeno-associated virus (AAV) based vectors for future gene therapies for the treatment of hereditary neuromuscular disorders (NMDs), we investigated the antibody levels in sera from patients with NMDs against 18 AAV types, including 11 AAVs with wild-type capsids, 5 AAVs with peptide-modified capsids and 2 AAVs with shuffled capsids. With regard to the wild-type capsid AAVs, the lowest binding antibody levels were detected against AAV6, AAV5, AAV12 and AAV9, whereas the highest binding antibody levels were detected against AAV10, AAV8, AAV1, and AAV2. The lowest neutralizing antibody levels against wild-type AAVs were detected against AAV12, AAV5, AAV9, AAV7, AAV8 and AAV10, and the highest neutralizing antibody levels were detected against AAV13, AAV2 and AAV3. Interestingly, the influence of peptide modifications or shuffling of AAV capsids on antibody binding and AAV neutralization seemed to depend on the parental AAV. While the sex of the serum donors had no significant impact on binding or neutralizing antibody levels, we observed a trend to higher binding antibodies in older serum donors against some AAV types and a clear positive correlation of neutralizing antibody titers with the age of the serum donors. The disease status on the other hand did not have a meaningful impact on antibody levels, with no changes in AAV neutralization. Our data indicate that several wild-type or peptide-modified AAV may be good candidates for therapeutic application due to low pre-existing antibody levels, and that the age of potential recipients rather than their health status with regard to NMDs has the biggest impact on vector applicability.

## Introduction

Adeno-associated viruses (AAVs) were initially discovered as a contamination in adenovirus preparations in 1965 (1, 2) and have been studied as viral vectors for over 30 years (3). They are non-enveloped, icosahedral, single-stranded DNA viruses belonging to the family of *Parvoviridae* with a genome of 4.7 kb and require helper viruses such as adenoviruses for productive infection and replication (4). Currently, there are at least 13 primate serotypes, hundreds of AAV variants and even more engineered AAVs (5–8), leading to a variety of opportunities for the choice and design of viral vectors in gene therapies (9). The choice of the AAV type for a particular application is guided mainly by the target cell tropism which differs widely across the different AAV types, and the level of pre-existing immunity against the AAV, which can be a particular concern for the systemic application of an AAV-based vector. Until now, seven AAV-based gene therapy treatments have received approval by the United States Food and Drug Administration (FDA) and the European Medicines Agency (EMA), and many more currently are or have been in clinical trials for a range of diseases including ocular, neurological, metabolic, hematological, neuromuscular and cardiovascular diseases and cancer (see (10, 11) for comprehensive reviews).

The development of gene therapeutic interventions is of great interest in the field of neuromuscular disorders (NMDs) (12, 13), which is a heterogeneous group of more than 100 disorders that affect different cellular populations of the neuromuscular axis including the motoneuron, peripheral nerves (Schwann cells and axons), the neuromuscular junction (pre-synaptic, synaptic and post-synaptic), as well as skeletal muscle cells. NMDs manifest in at least one of these cell types, and some diseases such as the neuromyopathies affect more than one of these cell populations (14). The first symptoms of most NMDs manifest in childhood, resulting in muscle weakness, myalgias and sensory symptoms. In most NMDs, symptoms become more severe as the disease progresses, leading to severe disability that can involve complete loss of muscle function and death through the loss of cardiac or respiratory function (15). To treat or even cure NMDs, gene therapy is a promising approach due to the lack of alternative therapy strategies and the fact that most NMDs are monogenic diseases, making them ideal candidates. The prevalence rates of individual NMDs are rare at 0.05 to 20 per 100,000 people (see (16) for a meta-analysis). Based on these prevalence rates, 300,000 patients are estimated to be affected by NMDs in Europe overall, making them an important target group for the development of novel treatment regimes. At the moment, only gene replacement therapies for the treatment of spinal muscular atrophy (SMA) (17) and Duchenne muscular dystrophy (18) have been approved, indicating the need for further investigations to possibly cure a large variety of NMDs. The AAV9-based gene therapy vector Onasemnogene abeparvovec (Zolgensma) has been approved for the treatment of SMA (19) by the FDA in 2019 and by the EMA in 2020, making it the first approved viral vector based gene replacement therapy for NMDs (17). More recently, the rhesus monkey AAVrh74-based gene therapy vector Delandistrogene moxeparvovec (Elevidys) has been approved for the treatment of Duchenne muscular dystrophy by the FDA in 2023 (18). More gene therapies for NMDs, all based on AAVs, are currently in the phase of pre-clinical animal models or even clinical trials (see (12, 13) for reviews), e.g. for the treatment of familial amyotrophic lateral sclerosis (ALS) (20), Pompe´s disease (21), alpha-sarcoglycan deficiency (22), familial limb-girdle myasthenia (23) or Charcot-Marie-Tooth disease (24). Due to their ability to transduce non-dividing tissues, their long-term *in vivo* transgene expression, their low potential of pathogenicity, as well as their replication deficiency (25), AAVs are the most promising viral vector candidates at the moment for gene therapy of NMDs.

High levels of pre-existing antibodies against AAV can prevent efficient gene transduction by neutralization of the vector, leading to a reduction or ablation of the therapeutic efficacy (26–29). While not resulting in elimination of the vector, also non-neutralizing AAV-binding antibodies can mediate undesired effects and lead to increased immune activation and liver toxicity and can contribute to complement activation and the development of thrombotic microangiopathies after systemic administration (30). Many clinical trials or approved therapies for ocular and neurological diseases use local administration of the AAV vectors into immunoprivileged sites such as the *corpus vitreum* or the brain, thereby circumventing the host immune response to a high degree. However, systemic application by intravenous infusion is the most common application route for gene therapy targeting metabolic, hematologic or neuromuscular diseases (11). It is therefore important to have a more detailed understanding of the prevalence of AAV-specific binding and neutralizing antibody levels in target populations. We therefore investigated the seroprevalence of 18 different AAV types in adult patients with different degenerative NMDs that are possible target diseases for future gene therapy (31). To be able to put antibody responses against AAV types into perspective, we did not focus solely on neuro- or myotropic AAV types but included other AAV types to obtain a broader picture of anti-AAV antibody responses.

## Materials and methods

### Ethics approval

The ethical approval for this study was granted by the Ethics Committee of the Medical Faculty of the Ruhr-University Bochum, under the reference number 20-6924. Serum donors gave written consent to their participation in the study and to the publication of the data derived from their contributions.

### Human samples

The serum samples used in this study were also tested previously for prevalence of antibodies against human adenoviruses (32). Briefly, 209 serum samples were collected at the University Hospital Bergmannsheil (Heimer Institute for Muscle Research; Ruhr University, Bochum, Germany) between August 2020 and March 2021. In total, 133 samples were obtained from adult patients with different NMDs and 76 from healthy volunteers at an age ranging from 17 to 97 years. The NMD patients were divided in seven subgroups: late onset Pompe’s disease (n=14), muscular dystrophies (n=25), degenerative motoneuron disorders (ALS; n=26), myotonic disorders (n=20), hereditary neuro(no)pathies (n=13), other genetic NMDs (n=24) and non-genetic NMDs (n=11). Inclusion criteria consisted of a solid molecular genetic or clinical diagnosis that were made by physicians specialized in NMDs and written consent to participate in the study. Exclusion criteria were the missing ability to give informed consent, an immunosuppressive therapy, an inflammatory myositis or known infections with hepatitis B or C virus or human immunodeficiency virus. All samples were centrifuged for 10 min and the sera were stored at –20°C.

### Cells

To accommodate different AAV types, multiple cell lines were used that were obtained commercially. The human embryonic kidney cell lines HEK293A (American Type Culture Collection (ATCC) # CRL-1573) and 293T (ATCC # CRL-3216) were propagated in Dulbecco’s modified Eagle medium (DMEM; Invitrogen-Gibco, Thermo Fisher Scientific, Karlsruhe, Germany) supplemented with 10% heat-inactivated fetal calf serum (FCS; FBS Superior, Sigma Aldrich, Taufkirchen, Germany), 50 μg/ml gentamicin (Thermo Fisher Scientific, Karlsruhe, Germany), and 20 μg/ml ciprofloxacin (Fresenius Kabi, Austria). The CHO-K1 cell line (ATCC # CCL-61) was propagated in Ham’s F-12 medium supplemented with 10% heat-inactivated FCS, 100 U/ml penicillin, and 100 μg/ml streptomycin (all Invitrogen-Gibco, Thermo Fisher Scientific, Karlsruhe, Germany). HepG2 cells (ATCC # HB-8065) were propagated in DMEM supplemented with 10% heat-inactivated FCS, 100 U/ml penicillin, 100 μg/ml streptomycin, and 2 mM L-Glutamine (Invitrogen-Gibco, Thermo Fisher Scientific, Karlsruhe, Germany). NIH 3T3 cells (ATCC # CRL-1658) were propagated in DMEM supplemented with 10% heat-inactivated FCS, 100 U/ml penicillin, and 100 μg/ml streptomycin. Cells were cultured in a humidified 5% CO_2_ atmosphere at 37°C.

### Viruses

Eighteen types of AAV-derived vectors were utilized, comprising eleven vectors harboring wild-type AAV capsids (AAV1, AAV2, AAV3, AAV5, AAV6, AAV7, AAV8, AAV9, AAV10, AAV12, and AAV13), five peptide-modified AAV capsids (AAV2P2, AAV2P4, AAV7P2, AAV9A2, and AAV9P1), and two shuffled capsid variants (AAVDJ and AAVLK03), each engineered to express luciferase.

**Figure 1:**
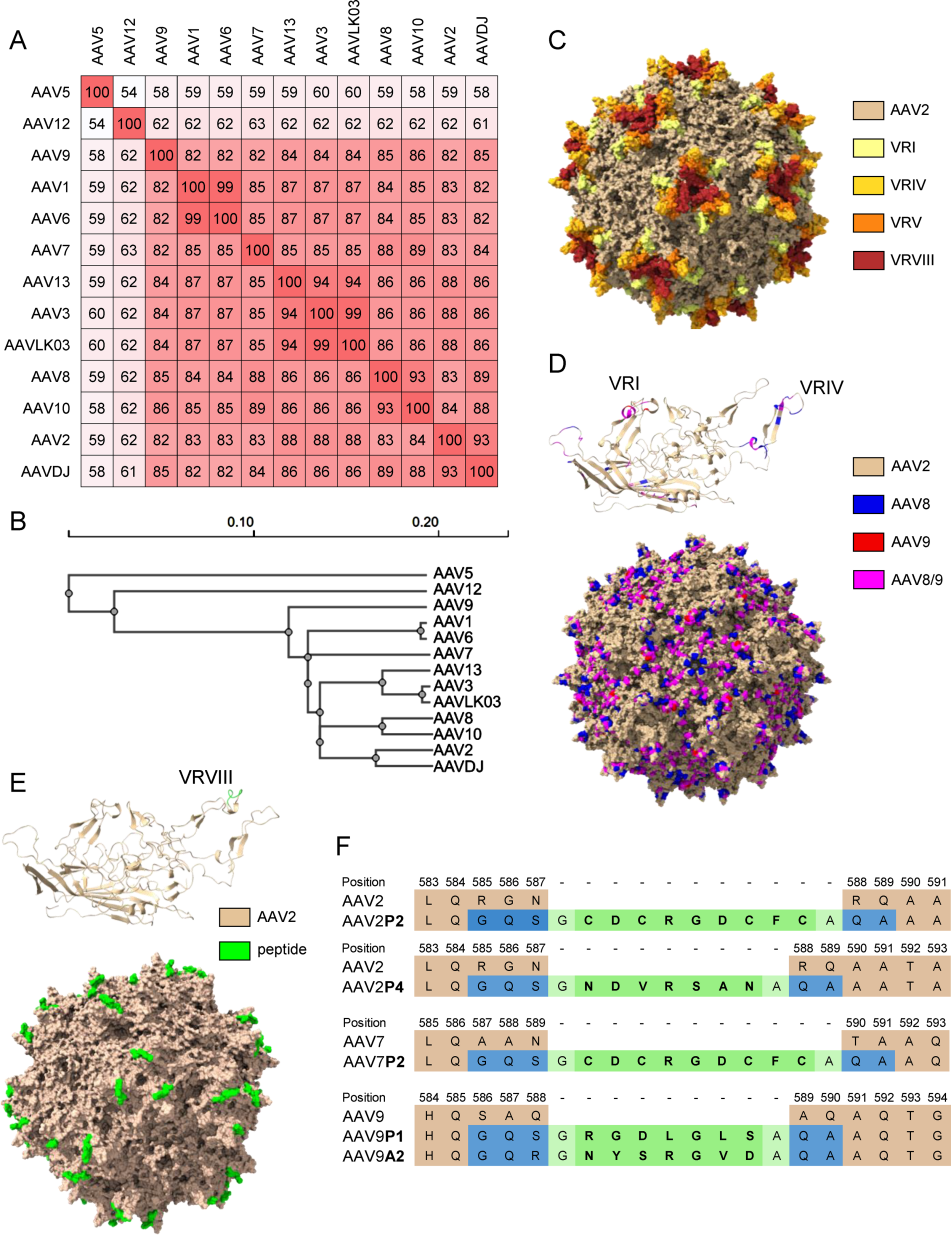
Sequence information for the AAV capsids used in this study. **(A)** VP1 amino acid sequence percentage identity matrix for all wild-type and shuffled AAV capsids used in this study and **(B)** guide tree of the same set of AAVs generated using Clustal Omega 1.2.4. **(C)** Visual representation of the AAV2 capsid structure [PDB ID 6IH9; (38)], the variable regions VRI, VRIV, VRV and VRVIII are highlighted in light yellow, yellow, orange and red, respectively. **(D)** Visual representation of the AAVDJ VP1 capsid protein (top) and whole capsid structure [bottom; PDBID: 7KFRM; (39)] with sequences derived from AAV2, AAV8 and AAV9 indicated by color. Wheat, blue and red indicate sequences from AAV2, AAV8 and AAV9, respectively, while magenta represents sequences that are identical in both AAV8 and AAV9. The labelled VRs in the VP1 structure represent the loops that were particularly divergent from AAV2. **(E)** Visual representation of peptide-modified capsids using the AAV2P2 VP1 capsid protein monomer (top) and the assembled capsid (bottom) as a representative example of the peptide-modified AAVs used in this study. The AAV2 sequence is shown as wheat colored, the inserted peptide sequence is colored in green. **(F)** Amino acid sequences surrounding the peptide insertion site in the peptide-modified AAV capsids used in this study. Wheat colored amino acids are the parental sequences, green colored amino acids are the inserted peptides. Blue colored amino acids resulted from the introduction of restriction enzyme cleavage sites to accommodate the peptide-encoding oligonucleotides, light green colored amino acids indicate glycine and alanine linker residues flanking all inserted peptide sequences.

The shuffled capsid AAVDJ has been obtained by shuffling of eight capsid sequences including AAV2, AAV8 and AAV9, and it has 93%, 89% and 85% capsid sequence homology to AAV2, AAV8 and AAV9, respectively ((33); **Figures 1A, B, D** and **Supplementary Figure S1**). The shuffled capsid AAVLK03 has been obtained by shuffling of the capsid sequences of AAV3, AAV4, AAV6, AAV8 and AAV9 (34), and it has 99%, 87%, 86% and 84% sequence identity to AAV3, AAV6, AAV8 and AAV9, respectively (**Figures 1A, B** and **Supplementary Figure S1**). The peptide modifications have been described before (35), and were achieved by insertion of 7 (P1, P4, A2) to 9 (P2) amino acids long peptide sequences into the variable region VIII of the capsid protein (**Figures 1E, F**). Predicted protein structures of the capsid protein VP1 and the whole capsid are shown for AAV2, for the shuffled capsid AAVDJ and for the peptide-modified AAV2P2 in **Figures 1C–E** with important capsid structures highlighted. A sequence alignment of the wild-type and shuffled AAV VP1 capsid protein sequences used in this study is shown in **Supplementary Figure S1**.

Production followed a standard triple-transfection technique with an AAV helper plasmid encoding the respective *cap* gene, and purification was achieved through iodixanol gradient density centrifugation. Please refer to (35) for more details on vector design and production.

### Binding antibody ELISA

For the detection of AAV-binding antibodies, 384-well Nunc Maxisorp plates (Sigma Aldrich, Taufkirchen, Germany) were coated with 2.5 x 10^7^ AAV particles in 25 µl coating buffer per well and incubated overnight at 4°C. After washing with PBS containing 0.1% Tween (0.1% PBS-T), plates were blocked with 75 µl PBS+20% FCS (FBS Superior, Sigma Aldrich, Taufkirchen, Germany) per well at room temperature for 5 hours. 25 µl of serum samples, diluted 1:1000 in PBS, were added to the wells and incubated overnight at 4°C. Following washing with 0.1% PBS-T, a horseradish peroxidase labeled polyclonal donkey-anti-human IgG antibody (Dianova, Hamburg, Germany) was applied. One-Step TMB Ultra (Thermo Fisher, Waltham, MA, USA) was used as the substrate, and the reaction was stopped by addition of 1N H_2_SO_4_. Absorbance at 450 nm was measured using a Mithras2 microplate reader (Berthold Technologies, Bad Wildbad, Germany).

For calculation of antibody concentrations, serial dilutions of purified human IgG (Sigma Aldrich, Taufkirchen, Germany) were coated to every plate and treated with blocking buffer, secondary antibody and substrate in the same way as the rest of the plates. No competition of FCS and bovine anti-AAV antibodies possibly present therein has been observed in comparison to other blocking reagents, while resulting in superior background reduction.

### Luciferase assay for detection of neutralizing antibodies

The titers of AAV-specific neutralizing antibodies were determined using a luciferase assay. First, serum samples were serially diluted 5-fold in DMEM, resulting in dilutions ranging from 1:10 to 1:31,250. These dilutions (25 μl/well) were mixed with AAV particles (10^7^ vector particles (vp)/25 μl/well) in a 96-well plate and incubated for 1 hour at 37°C. Subsequently, specific cell types (1×10^4^ cells in 50 μl medium/well) suitable for the respective AAV types were added (293A: AAV1, AAV2, AAV2P2, AAV7P2, AAV9A2, AAV9P1, AAV10, AAVDJ, AAVLK03; 293T: AAV2P4, AAV3, AAV6, AAV9, AAV13; CHO-K1: AAV5; NIH 3T3: AAV7, AAV8; HepG2: AAV12). After a 24-hour incubation at 37°C and subsequent removal of the culture medium, 100 μl of lysis buffer (30 mM Tris-HCl, 0.3 g/l dithiothreitol, 2% Ecosurf, and 20% glycerol) was added to each well. The plates were frozen at -20°C for 24 hours, thawed at room temperature, and 50 μl of the lysate was mixed with 25 μl of assay buffer (100 mM Tris-HCl, 5 mM MgCl2, 250 μM coenzyme A, 150 μM ATP, and 150 μg/ml D-Luciferin) per well. Each plate included five positive control wells each that were infected with 10^7^ vp/well or 10^6^ vp/well of the respective AAV without addition of any serum, and two negative control wells containing only cells, without serum and AAV. The analysis was conducted using a Mithras2 microplate reader. A detection value in an experimental well lower than the 10^6^ vp control value indicated that over 90% of the input virus had been neutralized, establishing the corresponding dilution factor as the neutralizing antibody titer of the serum sample.

### Sequence analysis and structure visualization

AAV VP1 capsid protein amino acid sequences of the wild-type and shuffled capsid AAVs used in this study were aligned using Clustal Omega 1.2.4 in the EMBL-EBI Job Dispatcher sequence analysis tools framework (36), and the alignment was colored using Jalview 2.11.3.3 (37). A percentage sequence identity matrix and guide tree were created in Clustal Omega 1.2.4. For structure visualization, the capsid structure of AAV2 [PDB ID 6IH9; (38)] and of AAVDJ [PDB ID 7KFR; (39)] were obtained from the RSCB protein data bank (40). Individual sequences were highlighted in UCSF ChimeraX 1.8 (41). The VP1 monomer structure of the peptide-modified AAV2P2 was predicted using SWISS-MODEL (42) using the AAV2 VP1 structure as a template. The assembled AAV2P2 capsid was generated from this predicted monomer structure using the VIPERdb v3.0 oligomer generator (43), the visualization with the highlighted peptide sequence was generated using UCSF ChimeraX 1.8 (41).

### Statistical analysis

The measured data was analyzed with the GraphPad Prism 8 software (GraphPad Software, La Jolla, CA, USA). For the analysis of binding and neutralizing antibody levels, the Kruskall-Wallis One-Way ANOVA on ranks was used for comparison of non-matched data and the Friedman non-parametric One-Way ANOVA on ranks was used for matched data, both together with Dunn´s multiple comparisons procedure. The Chi square test with Bonferroni adjustment for multiple comparisons was used for the analysis of neutralizing antibody prevalence. Correlation was computed by Spearman ranked correlation analysis.

## Results

We analyzed 133 serum samples from patients with NMDs and 76 serum samples from healthy individuals to identify promising viral vectors among 18 natural or engineered AAV variants based on low pre-existing seroprevalence of binding and neutralizing antibodies. As described before, the NMD patients were categorized into seven groups: late onset Pompe’s disease (n=14), muscular dystrophies (n=25), amyotrophic lateral sclerosis (n=26), myotonic disorders (n=20), hereditary neuro(no)pathies (n=13), other genetic NMDs (n=24), and non-genetic NMDs (n=11) (32). The cohort comprised 107 women and 102 men, with the NMD group including 62 women and 71 men, and the healthy group consisting of 45 women and 31 men. The average age was 52.2 years (range 17-92 years) for the NMD group and 60.1 years (range 19-97 years) for the healthy group.

**Figure 2:**
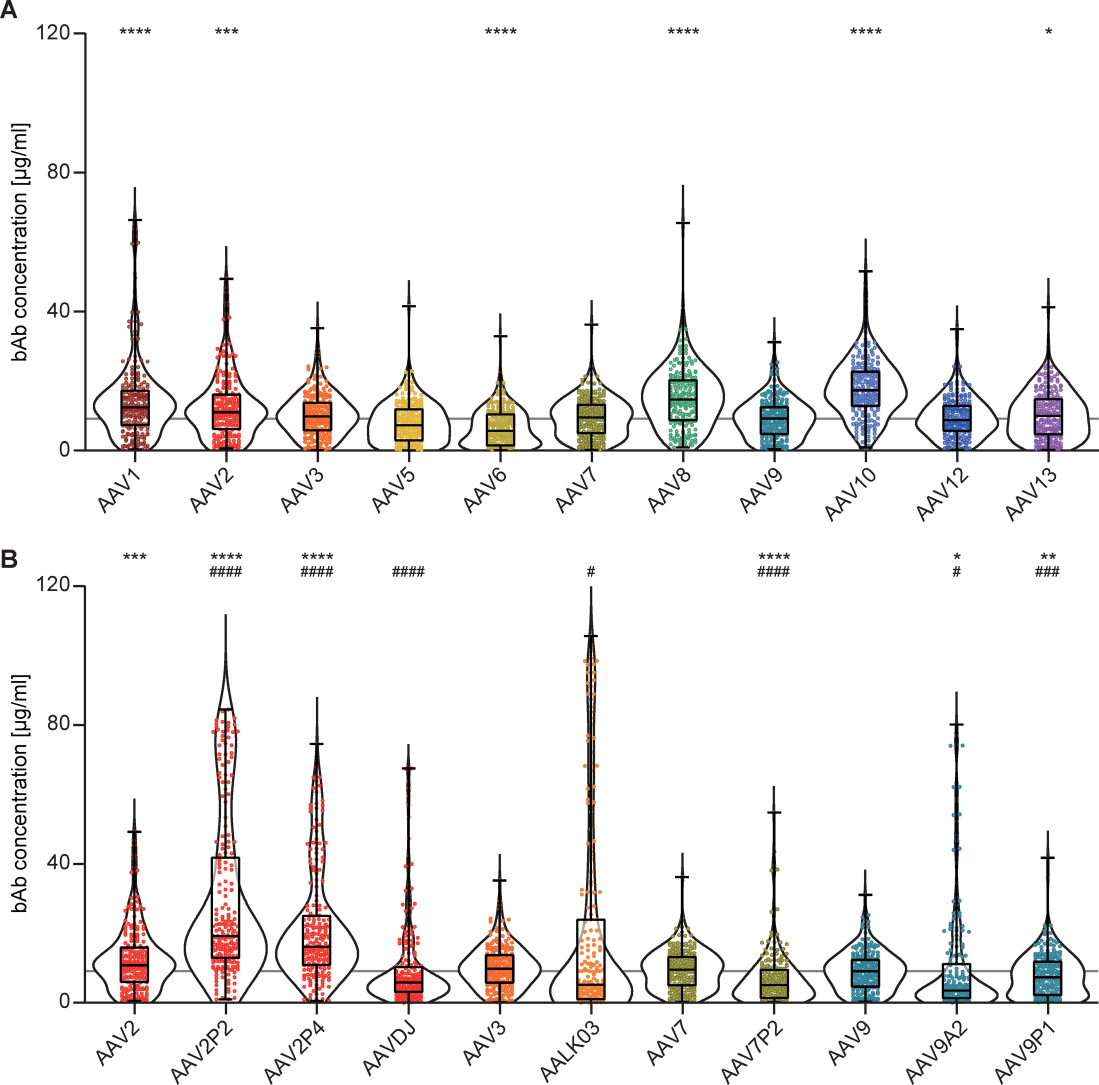
Binding antibody levels. All 209 serum samples of patients with NMD and of healthy controls were tested for binding antibodies (bAb) against the indicated AAV with wild-type capsids **(A)** or with peptide-modified or shuffled capsids **(B)**. Values shown in **(B)** for AAV2, AAV3, AAV7 and AAV9 are identical to **(A)** and shown for ease of reference. Each dot represents one individual sample, the boxes indicate the lower and upper quartile values with the line indicating the median value, the whiskers indicate the lowest and highest values, the violin plots indicate the data distribution. The grey line indicates the median bAb level against AAV9. Significant differences in binding antibody levels compared to AAV9 are indicated by asterisks (* *p* < 0.05; ** *p* < 0.01, *** *p* < 0.001, **** *p* < 0.0001; Friedman one-way ANOVA on ranks, Dunn´s multiple comparisons test), significant differences compared to the parental AAV are indicated by pound symbols (B; # *p* < 0.05, ### *p* < 0.001, #### *p* < 0.0001; Friedman one-way ANOVA on ranks, Dunn´s multiple comparisons test).

Binding antibody (bAb) levels exhibited broad variation across the 18 AAV types examined. The highest median bAb levels against wild-type AAVs were found against AAV10, AAV8, AAV1 and AAV2 (in descending order). Intermediate to low bAb levels were found for AAV3, AAV13, AAV7, AAV9, AAV12, AAV5 and AAV6 (in descending order, **Figure 2A**). Interestingly, peptide modifications of capsids and capsid shuffling had different effects on binding antibody levels compared to their parental AAV types (**Figure 2B**). While we detected significantly higher bAb levels against the peptide-modified AAV2P2 and AAV2P4 than against the parental AAV2, bAb responses against the shuffled capsid AAVDJ, which consists mostly of AAV2 but also of AAV8 and AAV9 sequences, were significantly lower than those against AAV2 as well as AAV8 but not AAV9. Contrary to what was observed for the peptide-modified AAV2, bAb levels against the peptide-modified AAV7P2 were significantly lower than against the parental AAV7. The shuffled capsid AAVLK03 that consists mainly of the AAV3 capsid showed a distinct bAb level pattern with many sera showing low reactivity and some others very high responses in comparison to the parental AAV3. While the peptide A2 and P1 modifications of the AAV9 capsid led to significantly lower bAb recognition compared to the parental AAV9, modification with peptide P1 showed a weaker effect for many of the serum samples. Overall, the bAb levels against AAV6, AAV7P2, AAV9A2 and AAV9P1 were significantly lower compared to AAV9, which can serve as a reference AAV type since an AAV9-based vector has already been approved for clinical use (17, 19).

**Figure 3:**
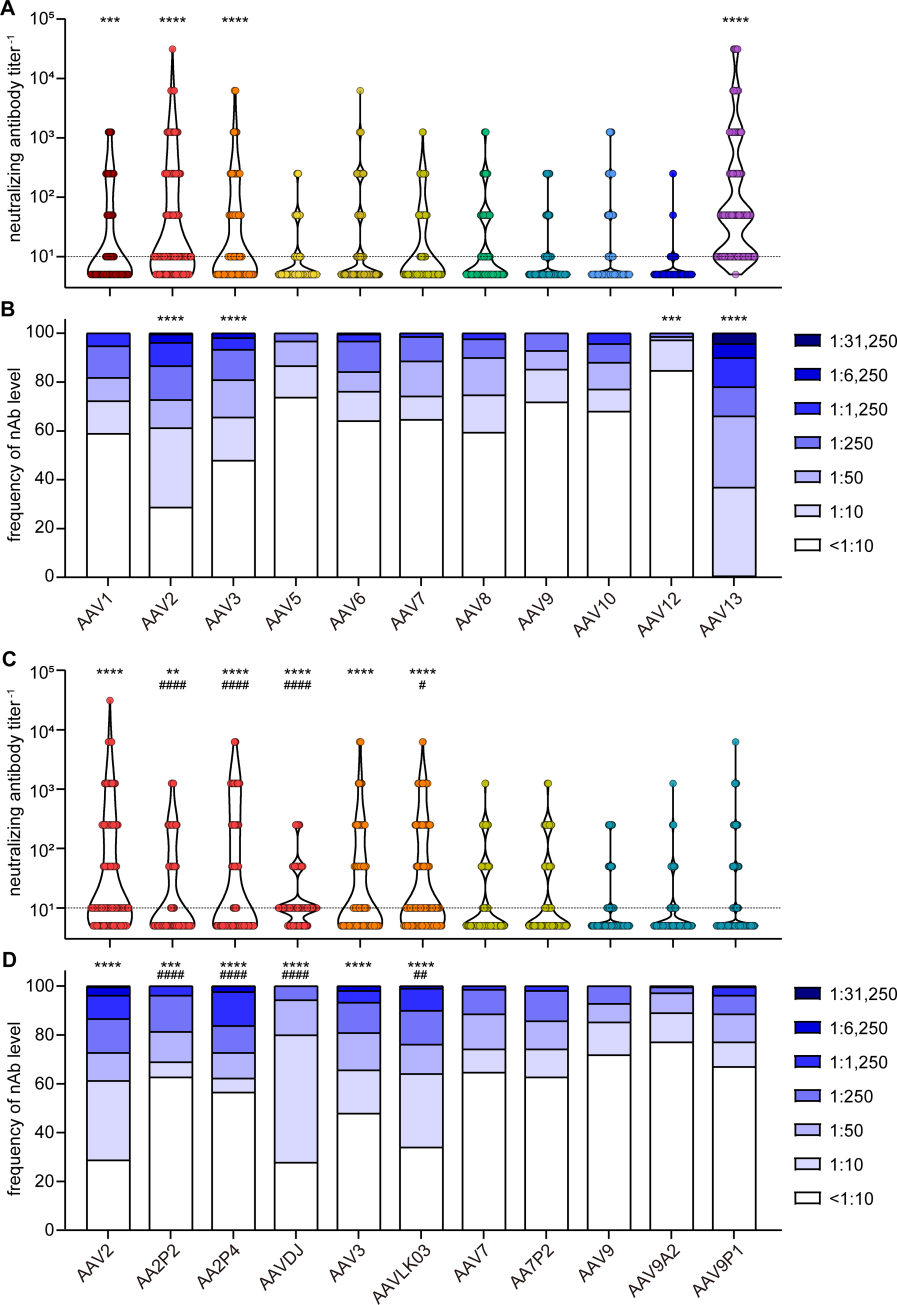
Neutralizing antibody levels and prevalence. All 209 serum samples of patients with NMD and healthy controls were tested for neutralizing antibodies (nAb) against the indicated AAV with wild-type capsids **(A, B)** or with peptide-modified or shuffled capsids **(C, D)**. Shown are individual nAb titers **(A, C)** and the frequency of nAb titers in the cohort **(B, D)**. Values shown in **(C, D)** for AAV2, AAV3, AAV7 and AAV9 are identical to **(A, B)** and shown for ease of reference. Each dot **(A, C)** represents one individual sample, violins indicate the data distribution, the dashed line indicates the detection limit. Significant differences in neutralizing antibody levels compared to AAV9 are indicated by asterisks [** *p* < 0.01, *** *p* < 0.001, **** *p* < 0.0001; Friedman one-way ANOVA on ranks with Dunn´s multiple comparisons test **(A)**, or Chi square test with Bonferroni adjustment for multiple comparisons **(B)**], significant differences compared to the parental AAV **(C, D)** are indicated by pound symbols [# *p* < 0.05, ## *p* < 0.01, #### *p* < 0.0001; Friedman one-way ANOVA on ranks with Dunn´s multiple comparisons test **(C)** or Chi square test with Bonferroni adjustment for multiple comparisons **(D)**].

When assessing neutralizing antibody (nAb) levels and prevalence (**Figures 3A, B**), we discovered that the highest neutralizing activity against wild-type AAVs was directed against AAV13, with only a single sample having no detectable nAb titer, and AAV13 neutralization was observed in the highest dilution of 1:31,250 for a few samples (4.4%). High neutralization levels were also observed for AAV2 and AAV3. Moderate to low neutralization levels were found against AAV1, AAV5, AAV6, AAV7, AAV8, AAV9 and AAV10, and prevalence of nAb against these AAV types ranged from 26% to 40%. The lowest prevalence was observed against AAV12 at approximately 15.3%.

The neutralizing antibody levels against the peptide-modified AAV2P2 and AAV2P4 were significantly lower than against the parental AAV2, whereas the neutralizing antibody levels against the shuffled capsid AAVDJ were significantly lower than against the parental AAV2 but significantly higher than against AAV8 and AAV9 (**Figures 3C, D**). For the other peptide-modified variants, we did not observe significant differences compared with the respective parental AAVs, whereas the nAb levels against AAVLK03 were slightly but significantly higher than against the parental AAV3.

**Figure 4:**
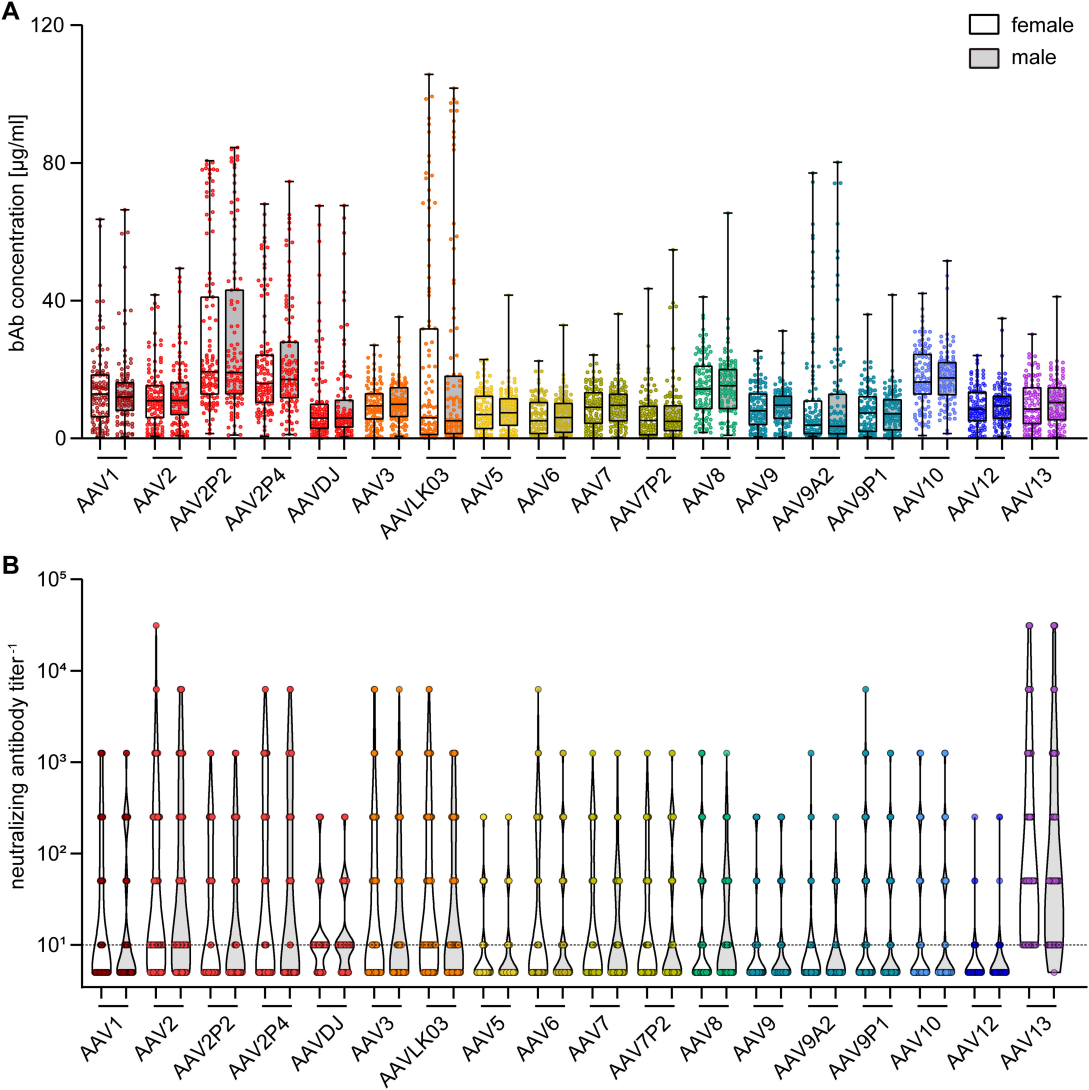
Binding and neutralizing antibody data stratified by sex of serum donors. The binding antibody levels **(A)** as shown in **Figure 2** and the neutralizing antibody levels **(B)** as shown in **Figure 3** were stratified by the sex of the serum donors, showing data of 107 female and of 102 male serum donors as indicated. The color guide for **(A, B)** is shown in the top right corner. Each dot represents one individual sample, the dashed line in **(B)** indicates the detection limit. No statistically significant differences in binding or neutralizing antibody levels of female and male serum donors were found using Kruskal-Wallis one-way ANOVA on ranks and Dunn’s multiple comparisons test.

**Figure 5:**
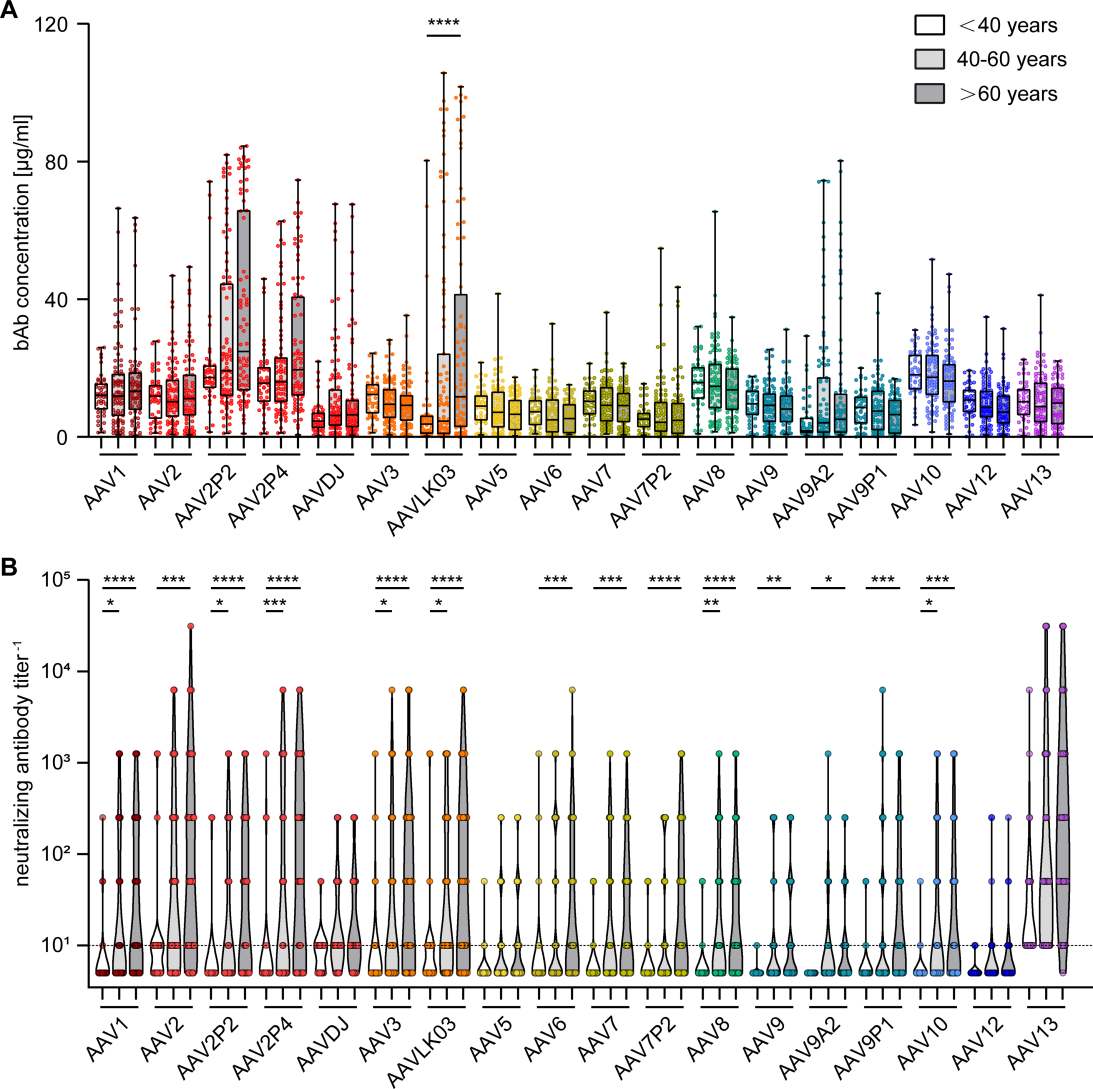
Binding and neutralizing antibody levels stratified by age of serum donors. The binding antibody levels **(A)** as in **Figure 2** and the neutralizing antibody levels **(B)** as in **Figure 3** were stratified by the age of the serum donors and divided in three groups: below 40 years of age (n=46), 40 to 60 years of age (n=87) and older than 60 years of age (n=76). The color guide for **(A, B)** is shown in the top right corner. Each dot represents one individual sample. The dashed line in **(B)** indicates the detection limit. Statistically significant differences in antibody levels compared to the age group “< 40 years” are indicated by asterisks (* *p* < 0.05; ** *p* < 0.01, *** *p* < 0.001, **** *p* < 0.0001; Kruskal-Wallis one-way ANOVA on ranks and Dunn’s multiple comparisons test).

To gain further insight into factors influencing AAV-specific antibody levels, we analyzed the impact of multiple factors: First, our data revealed that there were no significant sex-based differences in serum antibody levels, for both bAb and nAb levels (**Figure 4**). On the other hand, stratification of antibody data by age showed a clear age-related increase in both bAb and especially in nAb levels. Specifically, individuals over the age of 60 exhibited a trend to higher bAb levels against AAV2P2 and AAV2P4, and significantly higher bAb levels against AAVLK03, compared to those under 40 years of age (**Figure 5A**). More strikingly, the nAb levels showed a stark increase with age. The nAb levels in sera from donors older than 60 years were significantly higher against most tested AAVs than in sera from donors younger than 40 years, and they also showed an overall tendency to be higher in sera from donors aged between 40 and 60 compared to sera from donors younger than 40 years with significant differences observed for some AAV types (**Figure 5B**). Overall, we found a weak but significant correlation of nAb level and age for most of the tested AAV types (**Supplementary Figure S2**).

**Figure 6:**
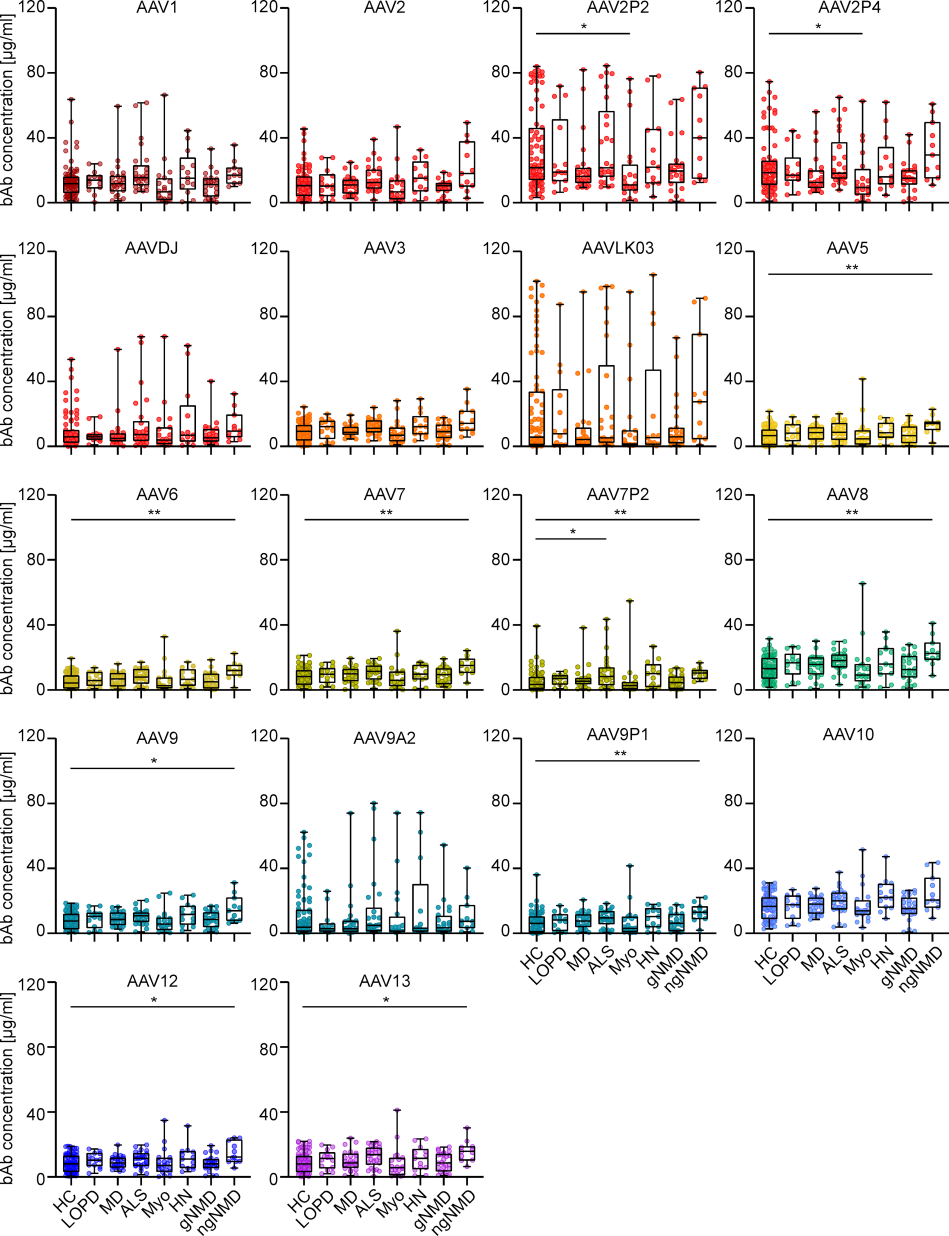
Binding antibody data stratified by health status of serum donors. The binding antibody levels as shown in **Figure 2** were stratified by the health status of the probands into eight groups: healthy controls (HC, n=76), late onset Pompe’s disease (LOPD, n=14), muscular dystrophies (MD, n=25), amyotrophic lateral sclerosis (ALS, n=26), myotonic disorders (Myo, n=20), hereditary neuro(no)pathies (HN, n=13), other genetic NMD (gNMD, n=24) and non-genetic NMD (ngNMD, n=11). Each dot represents one individual sample. Statistically significant differences in antibody levels compared to the group of healthy controls are indicated by asterisks (* *p* < 0.05; ** *p* < 0.01; Kruskal-Wallis one-way ANOVA on ranks and Dunn’s multiple comparisons test).

Furthermore, analysis of the effect of NMD status on AAV-specific antibody levels revealed that levels of both bAb and nAb across seven NMD groups were mostly comparable to those in the healthy control group, with only a few exceptions (**Figure 6** and **Supplementary Figure S3**). Sera from patients with myotonic disorders showed slightly but significantly lower bAb levels against AAV2P2 and AAV2P4 than sera from healthy controls, whereas sera from patients with ALS showed slightly higher bAb levels against AAV7P2 than sera from healthy donors, and sera from patients with other non-genetic NMDs showed slightly but significantly higher bAb levels against AAV5, AAV6, AAV7, AAV7P2, AAV8, AAV9, AAV9P1, AAV12 and AAV13 than sera from healthy controls (**Figure 6**). No significant differences in nAb levels were observed between sera from healthy individuals and from patients with NMDs (**Supplementary Figure S3**).

## Discussion

In this monocenter study, we investigated the seroprevalence of binding and neutralizing antibodies against eighteen AAV types including eleven wild-type capsid AAV types, five peptide-modified AAVs and two shuffled capsid AAVs in a cohort of patients with NMDs. Of note, we did not focus solely on neuro- or myotropic AAV types in order to obtain a broader picture of anti-AAV antibody reactivities. Our data clearly demonstrate different levels of pre-existing immunity against the different AAV types, with the lowest bAb levels observed against AAV9A2, AAV7P2, AAVLK03, AAV6, AAVDJ, AAV9P1 and AAV5, and the lowest nAb levels against AAV12, AAV10, AAV9P1, AAV9A2, AAV5 and AAV9, which may make these vector types particularly useful for future vector development and therapeutic application against NMDs. Our data are in concordance with other studies of AAV seroprevalence where high antibody levels against AAV1, AAV2 and AAV3, and low antibody levels against AAV5, AAV6, AAV7, AAV8, AAV9 and AAV12 have been reported before (44–49). The two shuffled capsid AAVs used in this study, AAVDJ and AAVLK03, have been described before to have low reactivity to the human immunoglobulin preparation IVIG, AAVDJ having been specifically selected for low IVIG reactivity (33, 34). While many of the wild-type and shuffled capsid AAVs tested in this study exhibit a high degree of sequence identity (**Figures 1A, B**), their sequence variability is higher in the variable regions (VRs; **Supplementary Figure S1**). The VRs are located on the outside of the AAV particles and are therefore more readily accessible for antibody binding, and are likely decisive for the differential antibody binding observed in our study (**Figure 1C**). Most of the binding antibody epitopes described in the literature are located within VRs [reviewed in (50)].

Of the wild-type AAVs tested in our study, AAV1, AAV2, AAV6, AAV7, AAV8, AAV9 and AAV12 have been described to have skeletal muscle tropism, AAV1, AAV6, AAV7, AAV8 and AAV9 to have cardiomyocyte tropism, and AAV1, AAV2, AAV5, AAV7, AAV9 and AAV10 to be neurotropic [for a review on AAV tropism, see (51)]. Many gene therapy clinical trials against a wide range of diseases including NMDs are currently ongoing [see (11) for a recent overview], and the first approved AAV based gene therapy for an NMD was the AAV9-based therapy for SMA with the drug Onasemnogene abeparvovec (17, 19), which has been very successfully applied in the past few years. The bAb level against AAV9 is an important consideration for eligibility of patients for the use of Onasemnogene abeparvovec, with an elevated bAb level of >1:50 defined as an exlusion criterion (19). Interestingly, bAb levels against the peptide modified AAV9A2 and AAV9P1 were even lower than against the parental AAV9 in our cohort. The peptide modifications have also been demonstrated to lead to differences in transduction efficiencies (35, 52), with AAV9P1 exhibiting a significantly increased transduction efficacy of the musculature after intravenous injection of mice that was clearly superior to the parental AAV9, which led to its alternative denomination AAVMYO (53). Taken together, these results suggest that this modified AAV9 may be similarly or even more effective for gene therapy of neuromuscular disorders, with more patients with NMDs being eligible for treatment due to reduced bAb reactivity. Interestingly, the peptide modification of AAV2 led to increased bAb reactivity against both AAV2P2 and AAV2P4, but at the same time the neutralization of these two modified AAVs was lower than the neutralization of the parental AAV2. The peptide P2 modification of AAV7, on the other hand, led to significantly reduced bAb levels against AAV7P2 compared to the parental AAV7, highlighting that the immunological effect of the peptide modification depends on the parental AAV and has to be determined experimentally. From this discrepant effect it can be concluded that the peptide P2 itself is not targeted by binding antibodies. Moreover, it can be speculated that the P2 surface modification, while interfering with neutralization, changes the capsid conformation in a way that makes other antibody epitopes more accessible, resulting in the increased antibody binding observed for AAV2P2 and AAV2P4. All peptide modifications of the AAVs used in this study have been achieved by insertions of the peptides into the variable region VIII within the capsid protein (**Figures 1C, E, F**), which protrudes from the capsid surface and is key to AAV-cell interactions, in order to modify the cell tropism and transduction efficiency (35). While it has been described that this region is also a frequently recognized antibody epitope, other regions of the AAV capsid have also been identified as anti-AAV antibody targets [reviewed in (50)]. A targeted modification of further antibody epitopes might lead to further reduction of anti-AAV reactivity, but would have to be evaluated for possible effects on target cell tropism and transduction efficiency. Since some peptide modifications of AAV2, AAV7 and AAV9, including AAV2P2, AAV7P2 and AAV9P1, have been shown to dramatically improve transduction of neural cells (52), these modified vectors may be important tools in the future for gene therapy of neurological and neuromuscular disease. The high bAb response to the P2-modified AAV2 may, however, preclude its systemic use since also non-neutralizing antibodies can effect undesired immunological outcomes after systemic administration and can contribute to the induction of thrombotic microangiopathy (30).

BAb and nAb levels in patients with NMDs were similar to healthy individuals, which is in accordance with the results obtained for anti-adenovirus antibody levels in this same cohort (32) and in spite of a reported higher occurrence of infections including respiratory tract infections in patients with NMDs compared to healthy individuals (54). As was also expected from the anti-adenovirus antibody study, we found the sex of the serum donors to be irrelevant for binding and neutralizing antibody levels. Furthermore, our data clearly showed a correlation of neutralizing antibodies with the age of the serum donors for most of the AAV types. Interestingly, this is in contrast to our previous study on the seroprevalence of human adenoviruses in the same cohort of patients with NMDs, where we did not observe a substantial increase of bAb or nAb levels with age (32). In other studies, it has been also demonstrated that AAV-specific antibody levels increased with age, with the lowest levels usually observed in young children after waning of maternal antibodies (44, 45, 55, 56). The increase of nAb with age is an important finding to consider for the application of AAV vectors as it indicates that the efficacy of AAV-based gene therapy may decline with the age of vector recipients. Of course, the rapid health deterioration observed with many NMDs is in itself a clear indication for a treatment at a young age. On the other hand, it was shown in a previous study of a cohort of 69 adult SMA patients aged between 20 and 58 years that only three sera exhibited elevated anti-AAV9 bAb titers >1:50 that would make them non-eligible for Onasemnogene abeparvovec treatment, and no correlation of anti-AAV9 bAb titers with the age of the patients was observed (57). Also in the study presented here, we still see low levels of bAb and nAb in many older serum donors against the lower-prevalent AAV types.

Our data showed a moderate to strong correlation of bAb and nAb levels for the high-prevalent AAV types AAV1, AAV2, AAV2P2 and AAV2P4 but also for the lower-prevalent AAV types AAV13, AAVDJ, AAVLK03, AAV7P2 and AAV9A2 (**Supplementary Figure S4**). It is interesting that while the bAb levels against AAV13 were similar to the bAb levels against AAV2, AAV3 and AAV7 and significantly lower than against AAV1, AAV8 and AAV10 (*p*<0.05), the nAb levels against AAV13 were the highest among all the AAV types tested, with only one donor showing a nAb titer below the detection limit. This may indicate a high neutralizing capacity of the AAV13-specific antibodies, or a certain degree of cross-neutralization by nAb induced by other AAV. The correlation of nAb levels between the tested AAVs was rather high for all AAVs with the exception of AAV12, and a strong correlation of bAb levels was also observed for some of the tested AAVs (**Supplementary Figure S5**), indicating that some of the observed AAV-specific antibodies were indeed exhibiting cross-reactivity.

There are several limitations to this study, including the fact that we only enrolled adult serum donors. According to previously reported findings it can be assumed that for most wild-type AAVs, the seroprevalence in children is lower than that observed in this cohort of adult patients (45, 55, 56). Furthermore, we only analyzed sera obtained in one study center in Germany, and regional differences in AAV seroprevalence have been shown before (47, 58). The seroprevalence of individual AAVs reported here can therefore not be generalized to the world-wide population, although we assume that the ranking of the different AAVs may be more generalizable. Finally, it may be pointed out that we analyzed the binding of IgG type antibodies in sera, and for some gene therapy applications, mucosal delivery of AAV vectors may be desirable. Notable examples for such an application are gene therapy approaches for the treatment of cystic fibrosis, where intrabronchial delivery of an AAV vector or aerosol administration to the lung have been evaluated in clinical trials (59–61). In the rabbit model, it has been shown that after repeated lung delivery of an AAV vector, serum antibody levels did not reflect antibody levels in bronchoalveolar lavage fluids, which remained undetectable (62). Therefore, it is reasonable to caution that for mucosal delivery, the level of secretory IgA antibodies in mucosal secretions and measured for example in bronchoalveolar lavage fluid would be of higher significance than serum antibody levels.

In conclusion, we found the age of the serum donors to be the main factor influencing the level of seroprevalence of AAV-specific antibodies, and we found similar binding and neutralizing antibody levels for patients with NMDs and healthy individuals. We found low binding and neutralizing antibody levels against many of the tested AAV, with the lowest overall responses against AAV5, AAV9 and AAV12 and the peptide-modified AAV7P2, AAV9A2 and AAV9P1. Our results suggest that many patients with NMDs would be eligible, also at an advanced age, for treatments with vectors based on these AAV types.

## Data Availability

The original contributions presented in the study are included in the article/**Supplementary Material**. Further inquiries can be directed to the corresponding author.
