# Supplementary material for: Seroprevalence of binding and neutralizing antibodies against 18 adeno-associated virus types in patients with neuromuscular disorders
Source: Front Immunol. 2024 Sep 27;15:1450858. doi: 10.3389/fimmu.2024.1450858 (PMC11466838; doi:10.3389/fimmu.2024.1450858)
Supplement: Supplementary file 1 [file DataSheet1.pdf]

Supplementary Figure S1

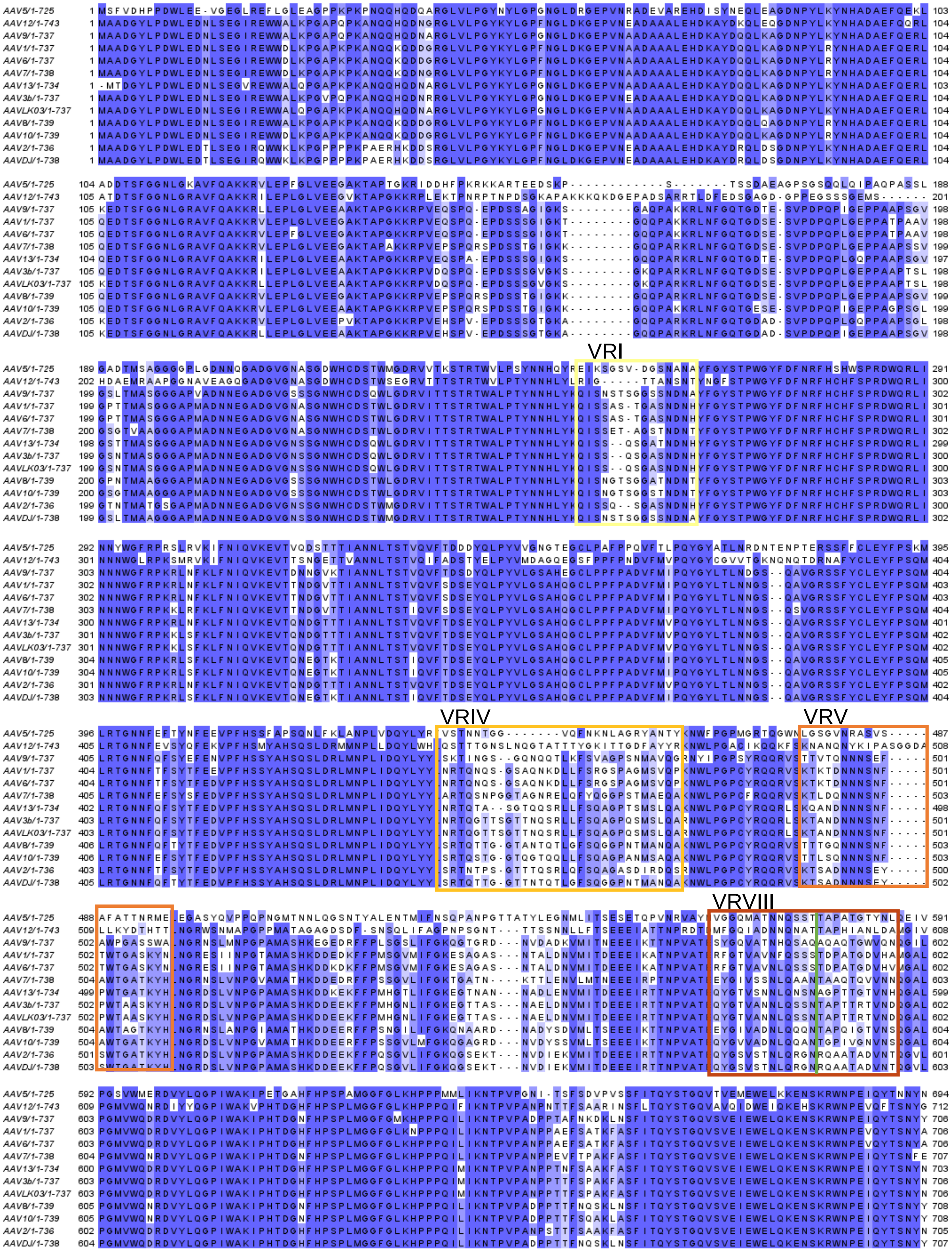

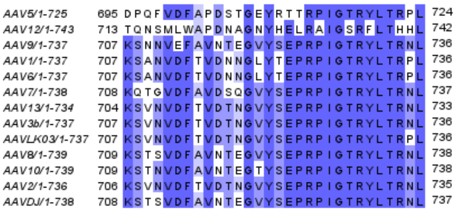

Multiple sequence alignment of wild-type and shuffled capsids used in this study. The alignments were generated using Clustal Omega 1.2.4, coloring is according to percentage identity and was generated in Jalview 2.11.3.3. Regions highlighted in light yellow, yellow, orange and red correspond to the variable regions I, IV, V and VIII, respectively. The green line represents the peptide insertion site in AAV2, 7 and 9.

Supplementary Figure S2

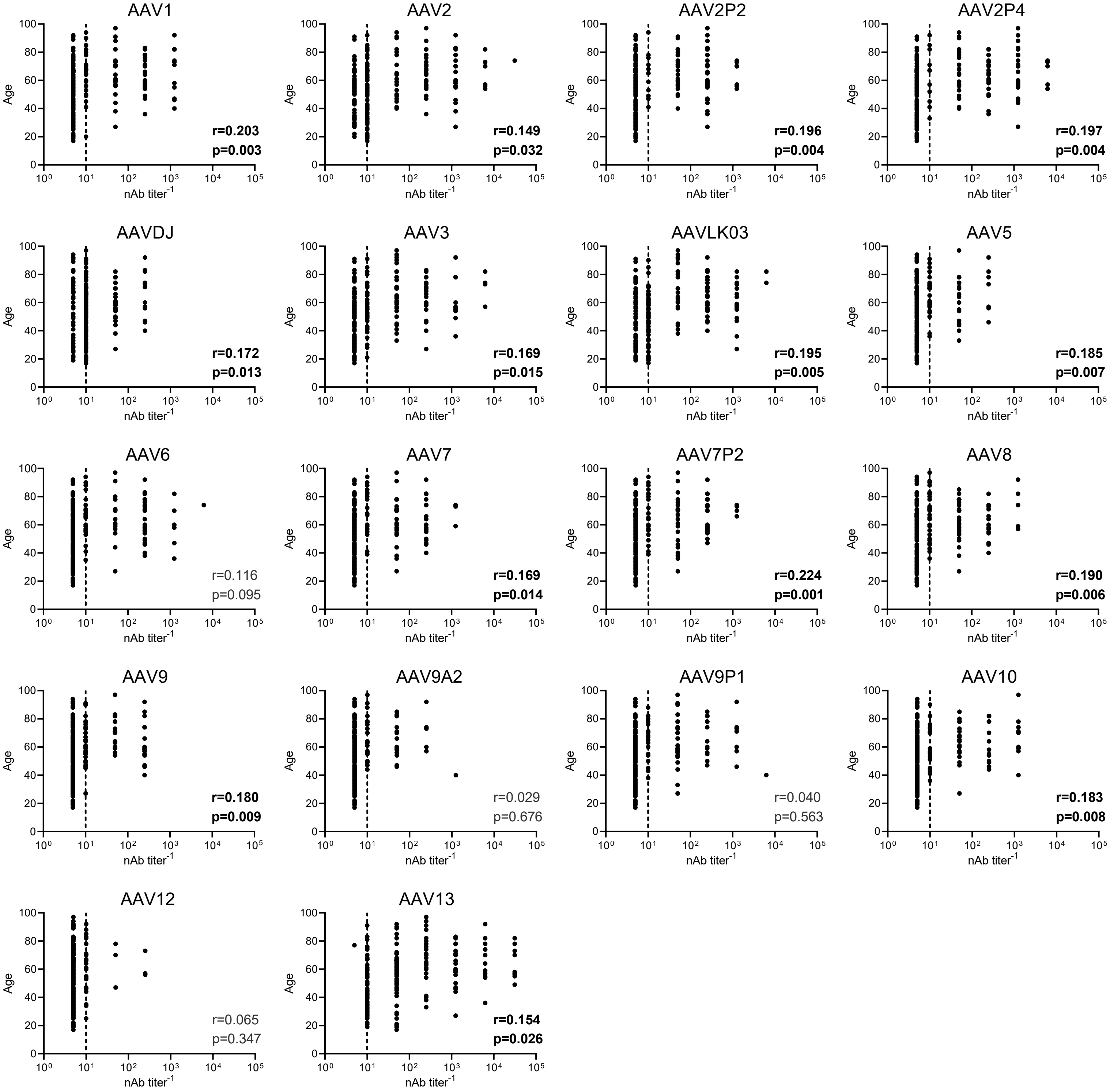

Correlation of AAV-specific nAb levels and age of the serum donors. The nAb titers against the indicated AAVs as shown in Figure 3 and the age of the serum donors were subjected to a Spearman ranked correlation analysis.
Each dot indicates an individual donor, the dashed line indicates the nAb detection limit.

Supplementary Figure S3

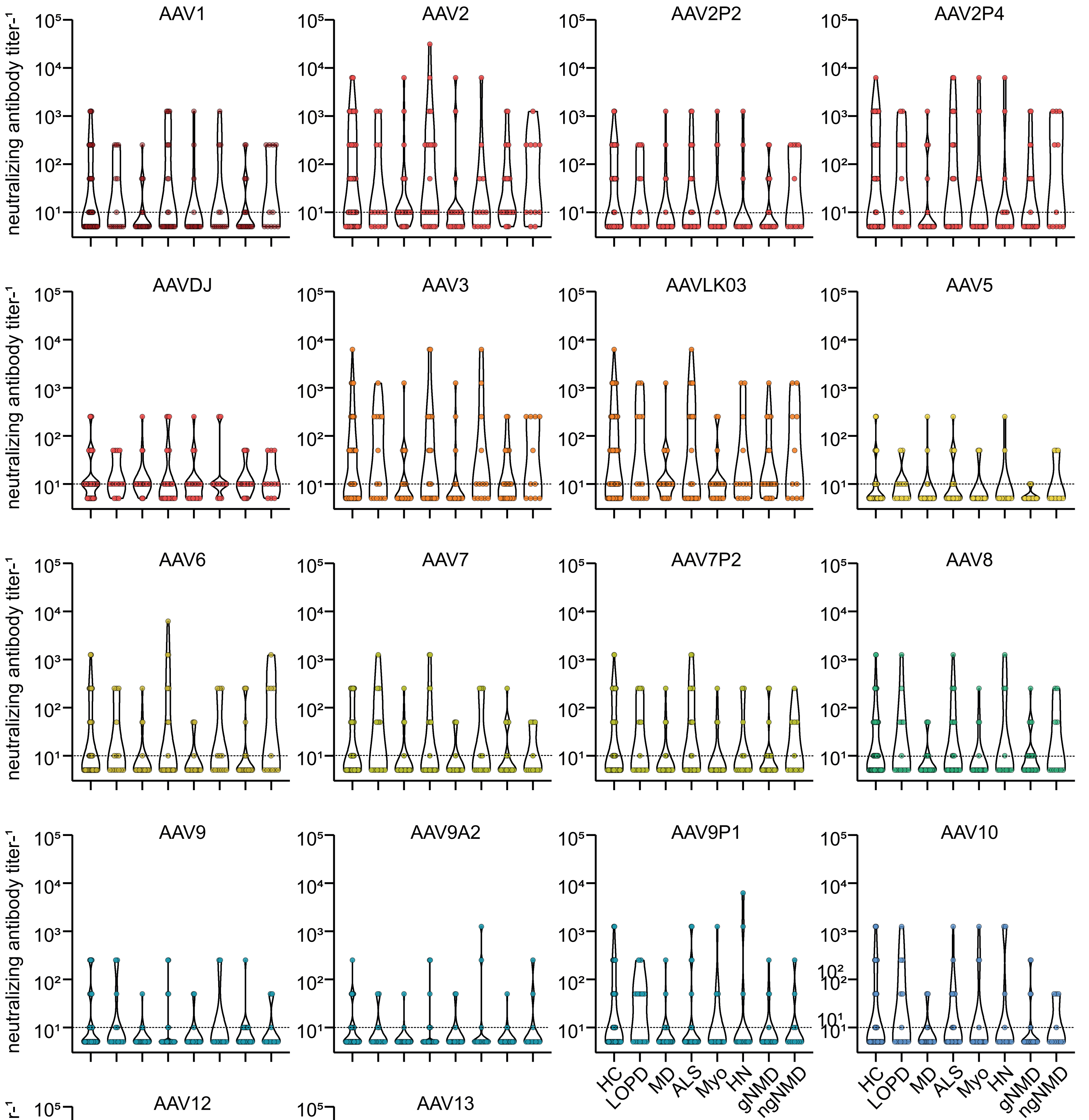

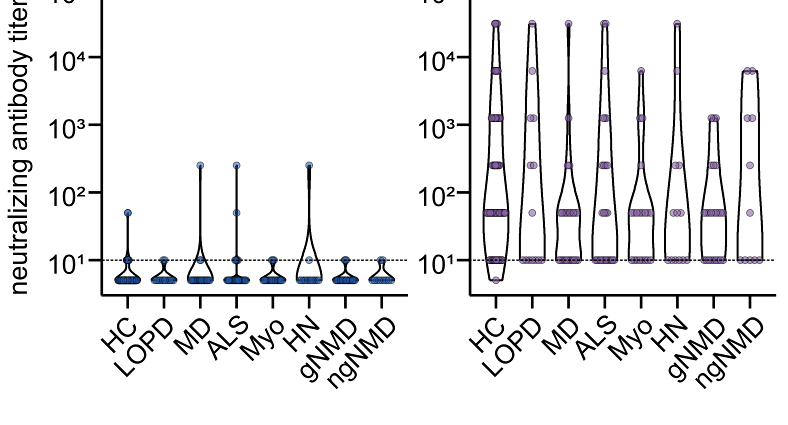

Neutralizing antibody data stratified by health status of serum donors. The neutralizing antibody levels as shown in Figure 3 were stratified by the health status of the probands into eight groups: healthy controls (HC, n=76), late-onset Pompe's disease (LOPD, n=14), muscular dystrophies (MD, n=25), amyotrophic lateral sclerosis (ALS, n=26), myotonic disorders (Myo, n=20), hereditary neuro(no)pathies (HN, n=13), other genetic NMD (gNMD, n=24) and non-genetic NMD (ngNMD, n=11).
The dashed lines indicate the detection limit, each dot represents one individual sample. No significant differences in antibody levels compared to the group of healthy controls were detected (Kruskal-Wallis one-way ANOVA on ranks and Dunn’s multiple comparisons test.

Supplementary Figure S4

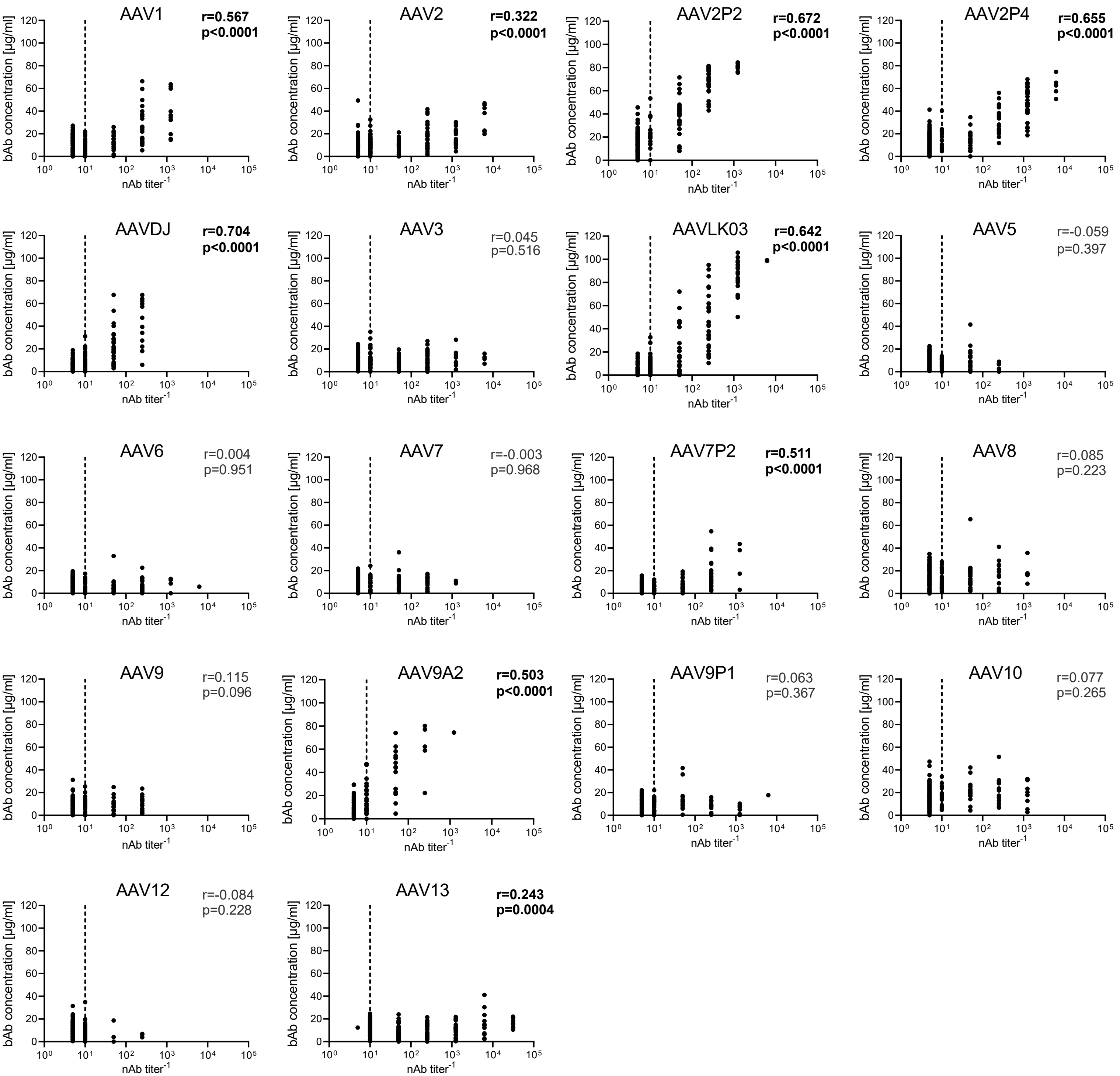

Correlation of AAV-specific bAb and nAb levels. The bAb titers as shown in Figure 2 and the nAb titers as shown in Figure 3 were subjected to a Spearman ranked correlation analysis.
Each dot indicates an individual donor, the dashed line indicates the nAb detection limit.

Supplementary Figure S5

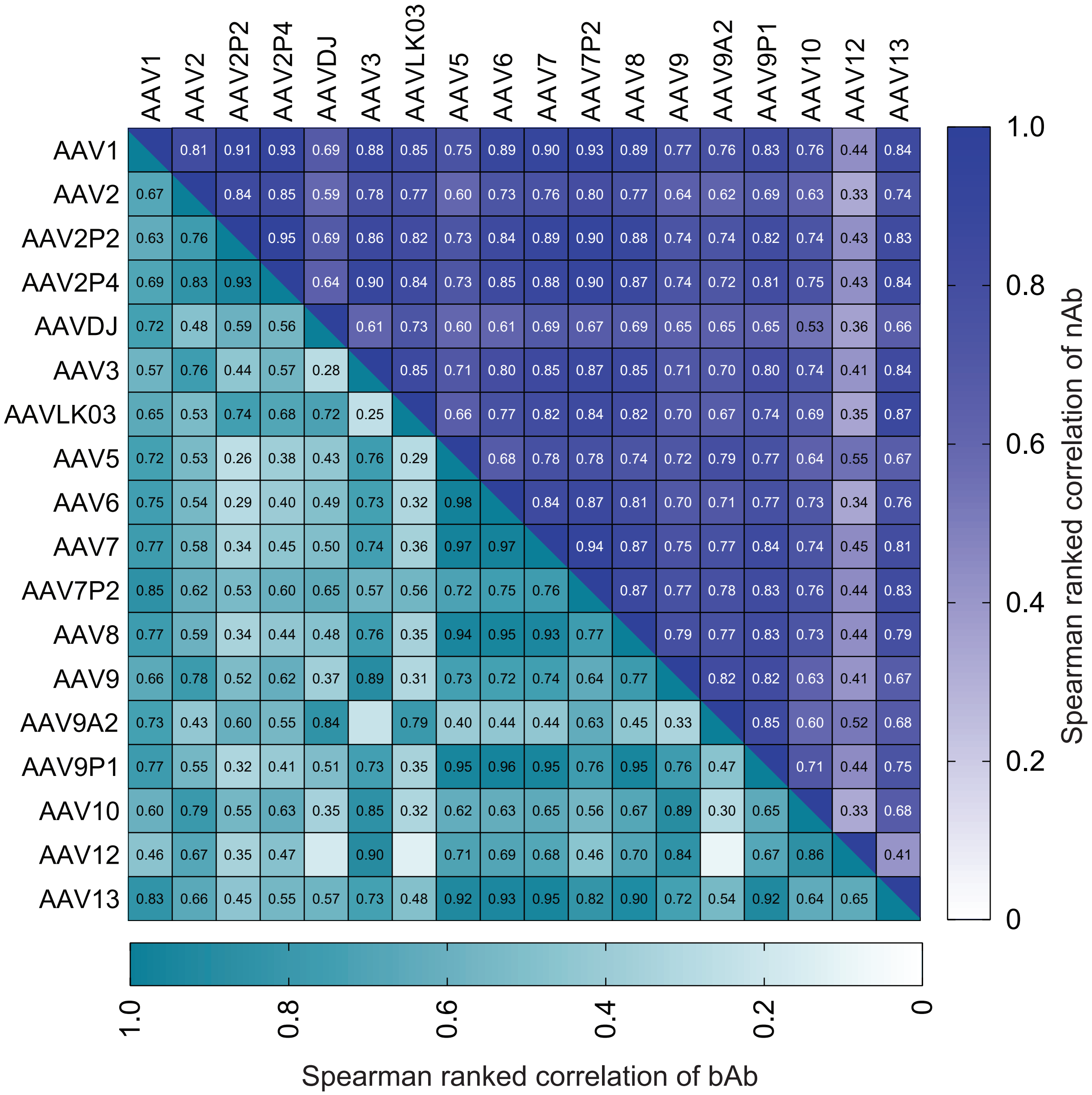

Correlation between bAb and nAb levels specific for different AAV types. A Spearman ranked correlation analysis was performed of the bAb levels as shown in Figure 2 (bottom left half-square) and of the nAb levels against the different AAV types as shown in Figure 3 (upper right half-square).
The colours indicate the correlation coefficient, values within the cells indicate the correlation coefficient if *p*<0.001.
